# Partial Trisomy 20qter on a Derivative Chromosome 22 in a Northern Italian Genetic Isolate with Oculo-Auriculo-Vertebral Spectrum-Related Features

**DOI:** 10.2478/bjmg-2026-0001

**Published:** 2026-10-14

**Authors:** M Imran, N Capodicasa, E Dida, M Bertelli

**Affiliations:** MAGI EUREGIO, 39100 Bolzano, Italy.; MAGI Balkans, Tirana 1019, Albania.; MAGI’S LAB, 38068 Rovereto, Italy.; MAGISNAT OMICS LLC, Atlanta Tech Park, Peachtree Corners, GA 30092, USA.

**Keywords:** copy-number gain, der chromosome 22, genetic isolate, *MYT1*, oculo-auriculo-vertebral spectrum-related features, subtelomeric rearrangement trisomy 20qter

## Abstract

Duplications of distal 20q are uncommon and the connection to oculo-auriculo-vertebral spectrum (OAVS) is still not clear. In this paper we describe a family-based cytogenetic case of a geographically isolated community in Northern Italy. Clinical data, pedigree information, and cytogenetic report from the proband were collected. Relatives were examined for OAVS-related features. Subtelomeric FISH analysis was performed with probes for 20qter and D22S1726 using peripheral blood lymphocytes to assess the rearrangement in detail. Descriptive statistics with 95% confidence intervals were computed. FISH demonstrated additional terminal material on der (22) consistent with a complex subtelomeric rearrangement and partial trisomy of 20qter. Among the 30 evaluated relatives, 15 (50.0%; exact 95% CI, 31.3–68.7) carried the rearrangement. Of these, 5 (33.3%; exact 95% CI, 11.8–61.6) exhibited features within the oculo-auriculo-vertebral spectrum, including preauricular tags, microtia or aural atresia, hemi-facial microsomia, ocular anomalies, vertebral defects, congenital heart disease, and hearing impairment. FISH does not provide information about the duplication segment or genes involved; therefore, *MYT1* inclusion into the duplication cannot be confirmed and requires further chromosomal microarray, SNP-array, MLPA or genome sequencing analysis. In this pedigree, the findings support the distal 20qter/20q13.33 as a possible candidate genomic region associated with OAVS-related features and support high-resolution genomic studies and genetic counselling in affected families.

## INTRODUCTION

Oculo-auriculo-vertebral spectrum (OAVS), known previously as Goldenhar syndrome and hemifacial microsomia, represents a heterogeneous group of congenital developmental syndromes characterized mainly by abnormal development of the structures derived from the first and second branchial arches. These abnormalities include preauricular skin tags, ear deformities, mandible hypoplasia, epibulbar dermoids, vertebral anomalies, hearing impairment, and others [1,2]. OAVS shows a genetic heterogeneity. Even if most cases stay genetically unexplained, there are reported contributors like rare sequence variants, chromosomal abnormalities, and pathogenic or likely contributory copy-number variants (CNVs). On the sequence-level side, candidates mentioned in OAVS, or in tightly related craniofacial microsomia phenotypes, include *MYT1*, *SF3B2*, *ZYG11B*, *EYA3*, *VWA1*, *ZIC3*, *AMIGO2*, and *PTCH2*, with different degrees of evidence. SF3B2 haploinsufficiency is viewed as one of the more common monogenic causes found for craniofacial microsomia and OAVS-related phenotypes, while *MYT1* variants seem rather rare. Recurrent, or again and again described, chromosomal/CNV findings feature changes involving 22q11.2, 14q22.3/14q23.1, including duplications in the *OTX2* region. Also, there are other uncommon imbalances, like 1p36/1p22, 5p13/5q, 10p14, 12p13 microdeletions, mosaic trisomies. Collectively, these points toward OAVS not being a single-gene condition, but more like a developmental spectrum involving multiple, dosage-sensitive routes, and cranial neural crest / branchial arch pathways [1,3,4,5,6,14,15,16,17,18].

*MYT1* gene, mapping to 20q13.33, continues to be a potential and biological candidate because rare *MYT1* sequence variants have been reported in OAVS. The functional role of *MYT1* in regulating craniofacial development controlled by the retinoic acid pathway has been shown experimentally [3,4,5]. In addition, a number of OAVS cases result from CNVs located in certain genes involved in craniofacial development [6]. Terminal 20q duplication represents a rather rare phenomenon; nevertheless, all of the available literature on the topic reports craniofacial, ear, palate, and cardiac anomalies similar to those observed in OAVS patients [7,8]. However, the current cytogenetic analysis does not prove that *MYT1* is duplicated in this case. Hence, for the current case report, the result can only be considered as a distal 20qter/20q13.33 regional gain rather than as evidence of *MYT1* duplication.

Chromosomal band 20q13.33-qter is linked to a specific set of anomalies occurring as a consequence of an excess number of copies of the region. Trisomy 20qter, despite its rarity (only about 23 cases known to date before 2014), represents a well-defined phenotype comprising craniofacial defects (microphthalmia, anteverted nares, large ears, cleft palate, micrognathia), heart malformations, and moderate developmental delay. In addition, the set of the aforementioned anomalies shows a high degree of consistency, while the level of morbidity varies widely [7,8,9]. Craniofacial abnormalities described above clearly overlap with the OAVS features, especially the ones related to the ears and mandible. At the same time, a deletion of the 20q13.33 chromosomal locus results in a completely different syndrome, characterized by developmental delay, speech defects, epilepsy, and autistic spectrum disorder with a series of candidate genes, namely *ARFGAP1*, *CHRNA4*, and *KCNQ2*. This example illustrates the bidirectional dose-dependent effect in this chromosomal region [8,10].

Isolated populations may help to detect rare genotype and phenotype correlations due to the founder effect, genetic drift, and lower heterogeneity of alleles in the population [11]. In the current report, we describe the terminal gain of the 20q locus on chromosome 22 observed in an extended family belonging to a Northern Italian genetic isolate presenting a variety of OAVS-like anomalies among its members. It should be noted that the purpose of the paper is not to provide evidence for pathogenicity of *MYT1* duplication, but rather to introduce a rare cytogenetic finding and outline the necessary studies in order to validate or invalidate this model.

## MATERIALS AND METHODS

The current short Communication is focused on a family and was based on available clinical charts, pedigrees, and cytogenetics reports of a large family descending from an isolated geographic region of Northern Italy. Supporting historical data confirmed longstanding geographic and demographic isolation; nonetheless, reconstruction of demographics was not considered the main purpose of this study.

The report was prepared from deidentified clinical, pedigree and cytogenetic information after consent for the publication had been obtained from living participants/legal guardians. Genetic counseling was provided in accordance with local standards.

The clinical examination focused on OAVS-related manifestations, including auricular dysmorphisms, facial asymmetry or hemifacial microsomia, eye defects, vertebrae problems, congenital heart disease, and hearing loss. Participants were classified as having OAVS-related features if they had at least one major OAVS manifestation within OAVS spectrum according to the clinical geneticists’ evaluations.

Chromosome analyses involved conventional G-banding and fluorescence in situ hybridization (FISH) on peripheral blood lymphocytes. The cytogenetics lab report stated that the analyses involved subtelomere probes for 20qter and D22S1726. According to the ISCN results, there was extra terminal 20q material located on der(22). Subtelomere FISH only detects extra terminal 20q material without providing any information about its size or gene content. For further refinement of break-points and identification of *MYT1* involvement, chromosomal microarray, SNP-array, MLPA or whole genome sequencing is needed.

Descriptive statistical methods were applied for the family. Estimates of carrier frequency and penetrance are accompanied by exact binomial 95% CI. These values were calculated as descriptive, pedigree-specific estimates only and were not intended to represent generalizable recurrence risks, given the limited family size and incomplete standardized phenotypic assessment of all relatives. The carrier-phenotype association test cannot be performed due to lack of standardized phenotypic data for all non-carriers. Figure 1 was created using R, specifically with the ggplot2, patchwork, grid, and readxl packages. Confidence interval values at 95% exact binomial level were calculated using binom.test, no identifiable laboratory image was used.

**Figure 1.**
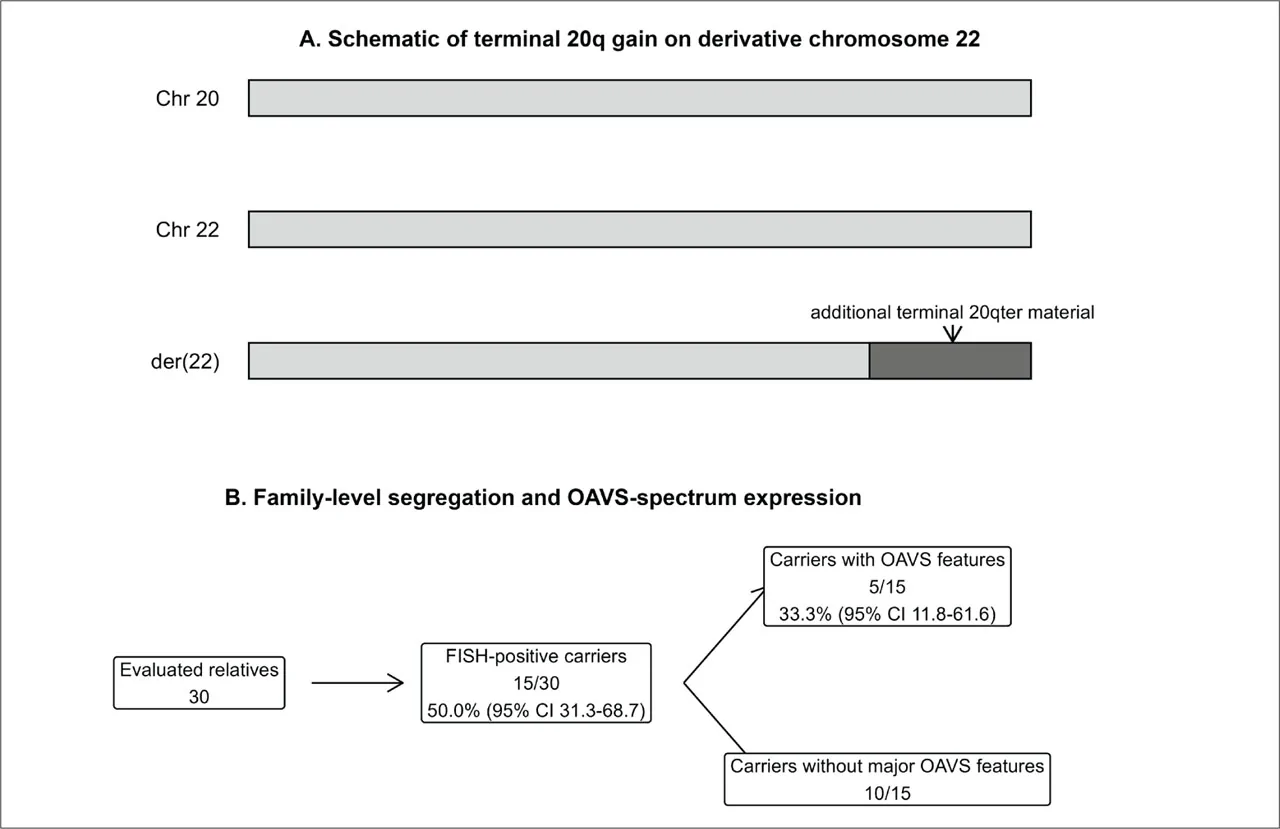
**Summary of cytogenetic and family-level analysis of terminal 20q gain on derivative chromosome 22 in relatives with OAVS-related features. A.** Illustration of the extra genetic material at the 20qter on the der (22) chromosome according to G-banding as well as subtelomere FISH using probes for 20qter and D22S1726. **B**. Family-level summary of individuals within the family carrying the rearrangement and exhibiting OAVS-related traits. Among 30 evaluated relatives, 15 had the rearrangement and five exhibited OAVS-related features. The duplicated interval and gene content, including whether *MYT1* is involved are unproven and require chromosomal microarray, SNP-array, MLPA, or genome sequencing.

## RESULTS

FISH also identified an additional signal of the terminal 20q segment on the derivative chromosome 22, with preservation of the D22S1726 locus, suggesting an unbalanced subtelomeric chromosomal rearrangement resulting in partial trisomy of the terminal 20q segment. The region duplication could not be delineated by FISH analysis only. The cytogenetic finding is summarized schematically in Figure 1A.

Of 30 family members analyzed, 15 had the chromosomal abnormality, with a carrier rate of 50.0% (95% CI, 31.3–68.7). Five carriers exhibited OAVS-related features, giving a pedigree-specific carrier penetrance estimate of 33.3% (95% CI, 11.8–61.6). The proportion of evaluated relatives with OAVS-related features was 16.7% (5/30, 95% CI, 5.6–34.7). Family-level carrier and phenotype summary is shown in Figure 1B.

Diverse clinical features were noted. They consisted of preauricular pits, microtia grade I–III, aural atresia, hemifacial microsomia, epibulbar dermoid or coloboma, cervical vertebral fusion/hemivertebra, closed ventricular septal defect repair, and hearing impairment **(Table 1)**. Clinical manifestations varied from isolated auricular defects to broader phenotypic overlap with OAVS, including craniofacial, ocular, vertebral, cardiac, and auditory findings.

**Table 1.** Deidentified Phenotype summary of carriers with OAVS-related features.

**Patient**	**Age**	**Sex**	**Auricular Anomalies**	**Facial Asymmetry**	**Ocular Findings**	**Vertebral Anomalies**	**Cardiac Defects**	**Other Features**
1	34 y	F	Bilateral preauricular tags, right microtia grade II	Hemifacial microsomia (right)	Epibulbar dermoid (right)	Cervical vertebral fusion C2–C3	None	Hearing loss (right)
2	28 y	M	Left preauricular tag, left microtia grade I	Mild hemifacial microsomia (left)	None	None	VSD (repaired)	Mandibular hypoplasia
3	19 y	F	Bilateral preauricular tags	Minimal facial asymmetry	None	Hemivertebra T4	None	None
4	42 y	M	Right microtia grade III, aural atresia	Severe hemifacial microsomia (right)	Coloboma (right)	Cervical vertebral anomalies	None	Hearing loss (right), cleft palate
5	15 y	F	Left preauricular tag	None	None	None	None	Hearing loss (right)

## DISCUSSION

The finding reported herein is clinically relevant, given that rare subtelomeric rearrangements may be difficult to interpret in isolated individuals. Familial cases of subtelomeric deletion and translocation are relatively common, and many large scales FISH studies on subtelomere region confirm that inherited abnormalities play an important role in causing subtelomeric abnormalities [12,13]. Within an extended family in a founder population, the same subtelomeric rearrangement may be seen in several relatives and provide more insight into the variable expressivity and penetrance estimate. In the current family, terminal gain of 20q was seen in several relatives and co-occurred with variable OAVS-related features, with prominent involvement of ear and mandible structures.

The most suitable interpretation is regional rather than gene-specific. The additional distal 20qter/20q13.33 material may contain one or more dosage-sensitive element(s) relevant to craniofacial and branchial arch development. *MYT1* located within 20q13.33 and has been reported as rare OAVS-associated gene involved in retinoic-acid related craniofacial development [3,4,5]. The proposed molecular mechanism remains speculative. Nevertheless, the FISH analysis confirms presence of extra terminal 20q material and cannot delineate the duplicated interval, breakpoints, and/or its gene content and regulation. Accordingly, *MYT1* should be viewed as only a positional candidate in this family, and its inclusion in the duplicated segment cannot be determined without chromosomal microarray, SNP-array, MLPA, or genome sequencing.

Variable expressivity is compatible with the wide spectrum of manifestations seen in OAVS cases. While some of the subjects demonstrated mild auricular deformities, other relatives showed a variety of abnormalities including facial, ocular, vertebral, cardiac or auditory abnormalities. Possible explanations include incomplete penetrance, genetic modifiers elsewhere in the genome, environmental factors or differential ascertainment of the cases. Thus, the penetrance estimate presented above must be regarded as a descriptive estimate for this particular pedigree only, as the data cannot be generalized to the entire population of OAVS because of the small sample size, selection criteria for the family on the basis of phenotype, and incomplete phenotype ascertainment for all relatives. Since the standardized evaluation of unaffected family members was incomplete, the results presented herein should be regarded as a carrier series rather than a definitive segregation or penetrance study.

Main limitations include small family-based sample size, potential ascertainment bias, incomplete standardized phenotypic assessment of all relatives, lack of detailed chromosomal mapping to identify breakpoints precisely, absence of experimental data concerning *MYT1* gene function, and incomplete evaluation of carriers for balanced translocations. Such drawbacks do not invalidate the study, but need to be taken into account in the interpretation. It seems fair to conclude that in the current family, distal 20qter/20q13.33 represents a candidate genomic region associated with OAVS-related features and requires further investigation using high resolution genomic studies.

## CONCLUSION

The gain of terminal 20q on derivative chromosome 22 was observed in several members of a Northern Italian genetic isolate with variable OAVS-related phenotypes. This indicates that distal 20qter/20q13.33 is a candidate chromosomal region in this pedigree; however, the duplicated interval and gene content, including whether *MYT1* is involved need verification via high-resolution genomic analysis.
